# Antimicrobial Postexposure Prophylaxis for Anthrax: Adverse Events and Adherence

**DOI:** 10.3201/eid0810.020349

**Published:** 2002-10

**Authors:** Colin W. Shepard, Montse Soriano-Gabarro, Elizabeth R. Zell, James Hayslett, Susan Lukacs, Susan Goldstein, Stephanie Factor, Joshua Jones, Renee Ridzon, Ian Williams, Nancy Rosenstein, the CDC Adverse Events Working Group

**Affiliations:** * Centers for Disease Control and Prevention, Atlanta, Georgia, USA; † New York Academy of Medicine, New York, New York, USA

**Keywords:** Anthrax, *Bacillus anthracis*, antimicrobial prophylaxis, adverse events, adherence

## Abstract

We collected data during postexposure antimicrobial prophylaxis campaigns and from a prophylaxis program evaluation 60 days after start of antimicrobial prophylaxis involving persons from six U.S. sites where *Bacillus anthracis* exposures occurred. Adverse events associated with antimicrobial prophylaxis to prevent anthrax were commonly reported, but hospitalizations and serious adverse events as defined by Food and Drug Administration criteria were rare. Overall adherence during 60 days of antimicrobial prophylaxis was poor (44%), ranging from 21% of persons exposed in the Morgan postal facility in New York City to 64% of persons exposed at the Brentwood postal facility in Washington, D.C. Adherence was highest among participants in an investigational new drug protocol to receive additional antibiotics with or without anthrax vaccine—a likely surrogate for anthrax risk perception. Adherence of <60 days was not consistently associated with adverse events.

Bioterrorist attacks involving the use of *Bacillus anthracis* in the fall of 2001 caused 22 cases of cutaneous and inhalational anthrax and placed many more persons at risk for this disease because of workplace exposures (1). The massive public health response to these events included an unprecedented prevention program in which approximately 10,000 persons across the eastern United States were offered >60 days of postexposure antimicrobial prophylaxis to prevent inhalational anthrax (2). We describe the exposed population and the provision of postexposure antimicrobial prophylaxis and analysis of data for associated adverse events and adherence to prophylaxis.

The large-scale use of antimicrobial prophylaxis to prevent anthrax within the setting of a bioterrorist attack has never been reported. While ineffective in killing B. anthracis spores, antibiotics are effective against replicating bacteria that develop from the spore following germination. After being inhaled, B. anthracis spores may not germinate immediately but can remain dormant in the lung and lymphatic system for weeks to months as they are slowly cleared by the immune system. As long as spores remain in the body, the risk of germination, replicating B. anthracis, and clinical anthrax exists. Based on initial risk assessments and the estimated efficacy of prophylaxis, antimicrobial postexposure prophylaxis was recommended during the 2001 anthrax outbreak (3,4).

Public health and military officials involved in bioterrorism preparedness initiatives had identified antimicrobial agents of choice for this purpose before the 2001 outbreak (5). Largely through the efforts of these officials, ciprofloxacin and doxycycline were approved by the Food and Drug Administration in 2000 and 2001, respectively, for use as antimicrobial prophylaxis to prevent anthrax and were offered as first-line agents to exposed persons (6,7). Because of safety concerns over the use of ciprofloxacin and doxycycline, amoxicillin, to which B. anthracis is known to be susceptible (8), was offered as prophylaxis to infants, children, and breastfeeding mothers, although it is not approved by the Food and Drug Administration for this indication (2).

In 2001, as soon as the risk for inhalational anthrax was identified, announcements were made recommending antimicrobial prophylaxis to exposed groups at risk; persons in these exposed groups were instructed to obtain prophylaxis from a central distribution point, where antibiotics were supplied from the National Pharmaceutical Stockpile (9). In December 2001, as vaccine became available, the Centers for Disease Control and Prevention offered persons who were recommended for 60 days of antimicrobial prophylaxis the opportunity to receive 40 additional days of antibiotics (ciprofloxacin, doxycycline, or amoxicillin), with or without three doses of anthrax vaccine, through an investigational new drug (IND) protocol. Exposed persons were encouraged to consult with their physicians regarding their individual risk for anthrax and the benefits of participation in the IND protocol (10).

## Methods

Antimicrobial prophylaxis campaigns were centered in six sites where persons were exposed: American Media, Inc. employees and visitors in Palm Beach County, Florida; workers and visitors at the United States Postal Service Trenton Processing and Distribution Center in Hamilton Township, New Jersey; employees and visitors at specific parts of the Hart Senate Office Building in Washington, D.C., as well as congressional mail workers who handled mail for that site; employees and visitors at the Brentwood postal facility in Washington, D.C.; employees working in selected areas of the Morgan postal facility in New York City; and workers and visitors with exposure to the Wallingford and Seymour postal facilities in Connecticut (2). Also among the cohort recommended for at least 60 days of antimicrobial prophylaxis were employees and visitors of the Department of State Annex 32 mailroom facility in Sterling, Virginia, and media workers associated with cutaneous cases in New York City. Ciprofloxacin was initially provided to all persons unless a specific contraindication existed. At the first and second refill visits at the New York City, New Jersey, Brentwood, and Connecticut sites (after antimicrobial susceptibility testing results were available), persons who had been taking ciprofloxacin were encouraged to change to doxycycline, provided no contraindications to doxycycline existed. Persons at the Hart Senate Building were provided a 60-day supply of ciprofloxacin during the first week that antimicrobial prophylaxis was distributed. In Florida, doxycycline was primarily provided at 30-day refill. At all sites, amoxicillin was provided to pregnant women, breastfeeding mothers, children, and some persons who had adverse events associated with ciprofloxacin and doxycycline.

### Data Collection

At each site, we used questionnaires distributed primarily at 10- and 30-day refill clinics to collect demographic, clinical, and adherence information. An adverse event was defined as any self-reported symptom while on antimicrobial prophylaxis. Respondents were asked to identify the antimicrobial agent taken most recently and select symptoms experienced while taking this agent from a list of possible adverse events. Early questionnaires used in the first antimicrobial prophylaxis campaign in Florida focused on the presence of a few specific symptoms and medical attention sought for adverse events. Later, in conjunction with 10-day refill clinics, a standardized questionnaire administered at the New Jersey, New York City, and Brentwood facilities collected information on a broader list of adverse events. We did not analyze 10-day New York City data because a large number of persons completed the questionnaires who had discontinued postexposure prophylaxis as recommended at 10-day follow-up. Modified versions of this initial questionnaire were used at 30 days at the Florida, New Jersey, Hart Senate Building, Brentwood, and New York City facilities. Questionnaires were self-administered in all sites except New Jersey, where they were administered by a health-care worker.

Potentially serious adverse events were identified based on adverse event data collected at 10- and 30-day follow-up (11,12). Persons who reported seeking medical attention because of adverse events associated with antimicrobial prophylaxis were further investigated. The definition of a serious adverse event, based on the Code of Federal Regulations (21 CFR 314.80), was applied to any of the following events associated with antimicrobial prophylaxis: death, life-threatening adverse event, inpatient hospitalization or prolongation of an existing hospitalization, persistent or substantial disability/incapacity, congenital anomaly/birth defect, or an important medical event that requires medical or surgical intervention to avert one of these outcomes. A clinician interviewed health-care providers and reviewed medical charts to assess the severity of the adverse events and determine whether they met the case definition. The relationship of the adverse event to the antimicrobial agent used was categorized as definite, probable, possible, remote, not related, and cannot assess. At day 30, a standardized data collection form was used.

### Program Evaluation after 60 Days

Beginning in late January 2002, after persons at each site had completed at least 60 days of antimicrobial prophylaxis, we evaluated the program for all persons in the exposed cohort. In our analysis, we included only persons who stated that they were recommended for at least 60 days of prophylaxis during the program evaluation interview. Through brief telephone interviews using a standardized questionnaire, we collected information on the ability of exposed persons to obtain antimicrobial prophylaxis and informational materials, associated adverse events, and adherence to prophylaxis. Adherence was defined as self-reported use of antimicrobial prophylaxis for at least 60 days. Respondents indicating the presence of adverse events were asked to identify their most severe or “single most serious” symptom, then identify other associated symptoms from a list of potential adverse events. Adverse events identified after the 60-day follow-up could be associated with overall use of antimicrobial prophylaxis, meaning respondents were attributing adverse events to one or more agents used as antimicrobial prophylaxis. Measures of perceived severity of symptoms, including whether medical attention was sought for adverse events, were included. Persons reporting nonadherence were asked to give the most important reason for not taking the antibiotic. We made multiple attempts to reach identified persons; follow-up to determine characteristics of nonrespondents is ongoing. Investigation of potentially serious adverse events reported after the 60-day follow-up is planned in a manner similar to prior serious adverse event evaluations.

### Data Analysis

 Statistical analyses were conducted by using SAS version 8 (SAS Institute, Inc., Cary, NC) statistical software. We used the χ^2^ test to compare proportions across each of the six sites; p<0.05 was considered statistically significant.

 We conducted two separate analyses for each of the six sites after 60 days using program evaluation data: one for nonadherence and one for occurrence of adverse events. The dependent (outcome) variable for the first analysis was nonadherence (nonadherence [1–59 days of antimicrobial prophylaxis] versus adherence [>60 days of antimicrobial prophylaxis]). The dependent (outcome) variable for the adverse event analysis was self-reported adverse events (a symptom reported versus no symptom reported). We excluded persons who reported not obtaining their prophylaxis or not taking any of it, as well as those for whom adherence information was not available. We constructed a logistic regression model for each dependent variable for each of the six sites. The independent (predictor) variables used in the logistic models included demographic and clinical variables from the 60-day program evaluation, including gender, age group, race, ethnicity, presence of adverse events, and participation in the IND protocol. Independent variables were retained in each of the site-specific models if their estimated parameters were statistically significant (p<0.05) in any model for the same dependent variable. We assessed colinearity and two-way interactions for all variables in each of the final multivariable models.

## Results

Approximately 10,000 persons were recommended for at least 60 days of antimicrobial prophylaxis to prevent inhalational anthrax. The largest number of persons on antimicrobial prophylaxis was associated with the Brentwood facility (n=2,743) and the smallest with the Hart Senate Building (n=600). We completed interviews on 6,178 persons; participation rates varied by site (Table 1).

**Table 1:** Response rates for persons recommended for at least 60 days of postexposure antimicrobial prophylaxis, 2001–2002

Anthrax investigation site	No. of persons prescribed prophylaxis^a^	Response rates for prophylaxis		
		10 days (%)	30 days (%)	60 days^b,c^ (%)
Florida	1,082	81	40	78
New Jersey	1,402	25	64	76
Hart Senate Building, Washington, D.C.	600	n/a^d^	59	82
Brentwood facility,Washington, D.C.	2,743	60	45	62
New York City	2,259	n/a	23	58
Connecticut	1,217	n/a	n/a	69

^a^When determining the number of persons prescribed prophylaxis, we excluded program evaluation respondents who indicated that they were not recommended for >60 days of antimicrobial prophylaxis. This number may vary from estimates using other data. ^b^Lists of persons in groups recommended for >60 days of antimicrobial prophylaxis were sometimes available only later in the campaign, so denominators may vary slightly for each collection period. ^c^No 60-day follow-up available on persons <18 years of age at time of 60-day evaluation. ^d^n/a, not available.

Most of the respondents were 40–64 years of age, and 60% were men. Of 2,444 women, 2% reported being pregnant or having been pregnant while taking antimicrobial prophylaxis. Median age was lowest at the Hart Senate Building site and highest at the Brentwood facility. Approximately 150 persons were <18 years of age at the start of the antimicrobial prophylaxis campaign; the Florida site had the most children (n=88). The number of children was estimated based on data collected at 10 and 30 days. Persons <18 years were not interviewed as part of the program evaluation after 60 days. Forty-one percent of respondents reported their race as white and 42% as African-American, but marked variation existed by site. Members of the Florida and Hart Senate Building cohorts were primarily Caucasian, while persons at Brentwood facility were primarily African-American (Table 2).

**Table 2:** Demographic data for persons recommended for at least 60 days of postexposure prophylaxis, 2001–2002

Characteristic	Florida (%) (n=780)	New Jersey (%) (n=1,061)	Hart Senate Building, Washington, D.C. (%) (n=485)	Brentwood facility, Washington, D.C. (%) (n=1,694)	New York City (%) (n=1,315)	Connecticut (%) (n=843)
Male	62	66	59	58	55	67
Pregnant	2	1	3	2	2	3
Caucasian	84	63	76	5	17	63
African-American	4	23	16	87	45	21
Median age (yrs)	40	46	34	51	46	44
Age range (yrs)	(17–86)	(18–77)	(17–75)	(19–79)	(18–78)	(17–85)

Almost all (97%) respondents obtained an initial supply of antimicrobial prophylaxis. Three percent (n=182) of respondents reported difficulty in obtaining their supply of prophylaxis, and of these, most (83%) were able to get 60 days of prophylaxis. Ten percent of respondents took no antimicrobial prophylaxis, although they collected an initial supply. This group and those who never obtained antimicrobial prophylaxis compose the overall group of 787 respondents who reported not taking any of their prophylaxis. Forty-eight respondents did not provide any adherence information. Persons who took at least one dose of antimicrobial prophylaxis numbered 5,343 (86%); fewer than half of these respondents took only one agent as antimicrobial prophylaxis. Fifty-nine percent of respondents taking at least one dose of antimicrobial prophylaxis (n=3,156) took two antimicrobial agents as prophylaxis; 56% (n=2,984) took ciprofloxacin for one part of their course and doxycycline for the rest. Data from 10, 30, and post-60 days show an overall shift in the most recent antimicrobial agent used from ciprofloxacin (84% at day 10) to doxycycline (61% at day 60).

### Adverse Events

 Of the 5,343 persons who reported taking at least one dose of antimicrobial prophylaxis, 57% (n=3,032) reported adverse events during the first 60 days of antimicrobial prophylaxis use. Reporting of adverse events varied by site, ranging from 42% of respondents at the Connecticut facility to 65% at the Brentwood facility. Thirty-two percent of respondents with adverse events reported diarrhea or stomach pain with their most recent antibiotic, 27% nausea or vomiting, 25% headache, and 22% dizziness. The most commonly reported categories of symptoms were gastrointestinal (44%, including nausea or vomiting, diarrhea or stomach pain, heartburn, and pain with swallowing) and neurologic (33%, including headache, dizziness, lightheadedness, fainting, and seizure). Of the 3,032 persons reporting at least one adverse event, 23% identified “diarrhea or stomach pain” and 19% “nausea or vomiting” as their “most serious” symptom. Among persons reporting adverse events, 17% graded them as severe, 45% as moderate, and 41% as none/mild. Twenty-six percent of persons with adverse events reported missing at least 1 day of work because of symptoms.

 At 10 days, the rate of one or more adverse events among persons taking ciprofloxacin most recently (45%) did not differ significantly from that of persons taking doxycycline most recently (49%). At day 30, this rate was slightly higher (77%) among persons taking ciprofloxacin most recently than persons taking doxycycline most recently (71%, p<0.01) (Table 3).

**Table 3:** Adverse events at 10 and 30 days, by most recent antimicrobial agent, all sites,^a^ 2001–2002

Adverse events	Day 10			Day 30		
	Ciprofloxacin (%) (n=2,446)	Doxycycline (%) (n=165)	p value	Ciprofloxacin (%) (n=737)	Doxycycline (%) (n=2,050)	p value
>1 adverse event	45	49	0.27	77	71	<0.01
Gastrointestinal symptoms (nausea, vomiting, diarrhea, abdominal pain, or heartburn)	26	26	0.89	42	49	<0.01
Fainting, dizziness, light-headedness, or seizuresb	18	11	0.08	23	18	0.01
Rash, hives, or itchy skin	7	7	0.8	14	14	0.6
Joint problems^b^	8	7	0.6	25	16	<0.01

^a^Day 10 data includes the Florida, New Jersey, and Washington, D.C., Brentwood sites; Day 30 data includes the Florida, New Jersey, Washington, D.C., Hart Senate Building , and New York City sites. ^b^Day 10 reports of these symptoms not collected at the Florida site.

Univariate analysis of factors associated with the presence of adverse events showed male respondents were less likely to report adverse events than were female respondents in all sites except Connecticut. Compared with the youngest age group, persons who reported adverse events were less likely to be >65 years of age. Persons with adverse events were significantly more likely to enroll in the IND protocol in the Brentwood facility (Table 4).

**Table 4:** Univariate analysis of factors associated with adverse events, post 60-day program evaluation data, 2001–2002^a^

Variable	Reports of adverse events among persons taking at least one dose of prophylaxis												
	Florida (n=744)		New Jersey (n=1,028)		Hart Senate Building, Washington, D.C. (n = 472)		Brentwood facility, Washington, D.C. (n=1,619)		New York City (n=882)			Connecticut (n=598)	
	(%)	p value	(%)	p value	(%)	p value	(%)	p value		(%)	p value	(%)	p value
**Gender**													
Male	50	<0.01	54	<0.01	54	0.01	60	<0.01		44	<0.01	41	0.53
Female	63	Ref	68	Ref	65	Ref	74	Ref		61	Ref	44	Ref
**Age group**													
17–39 yr	55	Ref	58	Ref	61	Ref	69	Ref		56	Ref	40	Ref
40–64 yr	55	0.99	59	0.75	53	0.10	66	0.38		51	0.20	44	0.37
>65 yr	41	0.19	39	0.03	40	0.34	52	0.04		37	0.04	19	0.10
**Race**													
African-American	54	0.88	61	0.35	46	0.02	67	0.36		52	0.74	36	0.14
All others	55	Ref	58	Ref	61	Ref	63	Ref		51	Ref	44	Ref
**Ethnicity**													
Hispanic	55	1.00	60	0.87	44	0.38	57	0.38		56	0.12	39	0.65
Non-Hispanic	55	Ref	59	Ref	59	Ref	66	Ref		50	Ref	42	Ref
**IND enrollment**													
Yes	48	0.29	61	0.34	68	0.11		<0.01		56	0.40	50	0.13
No	56	Ref	57	Ref	57	Ref	63	Ref		51	Ref	41	Ref
**Received printed materials about adverse events**													
No	70	0.03	61	0.73	73	<0.01	78	0.02		29	0.01	53	0.36
Yes	54	Ref	58	Ref	54	Ref	66	Ref		53	Ref	42	Ref

^a^IND, investigational new drug; Ref, referent.

Multivariable analysis also showed that persons reporting adverse events were less likely to be male in all sites except Connecticut. At the Hart Senate Building site, persons with adverse events were less likely to be African-American. At the Brentwood site, persons with adverse events were more likely to have enrolled in the IND protocol.

### Medical Attention for Adverse Events and Serious Adverse Events

Of 2,907 persons participating in 10-day follow-up, 7% reported seeking medical attention. Follow-up at 10 days for serious adverse events in the Florida, New Jersey, and New York City facilities found no hospitalizations attributable to antimicrobial prophylaxis in persons seeking medical care for symptoms consistent with anaphylaxis (difficulty breathing, rash or itchy skin, throat tightness, or lip and tongue swelling) (11). Of 3,374 persons participating in 30-day follow-up, 13% reported seeking medical attention. Of 2,135 persons with follow-up information available at 30 days in the Florida, New Jersey, New York City, and the Hart Senate Building facilities, seven persons (0.3%) were found to have had a serious adverse event, including three persons hospitalized. Ten- and 30-day follow-up data were not available for Connecticut. Four persons had reactions in which the relationship to antimicrobial prophylaxis was judged to be definite or probable, while the remaining three were classified as not related or could not assess. Two of four serious adverse events with a definite or probable relationship to antimicrobial prophylaxis were characterized by diffuse rash and systemic symptoms; the remaining two involved swelling of the face and neck. Two persons were treated as outpatients, one was treated in the emergency department, and the remaining patient was briefly hospitalized. All four recovered without sequelae.

At the post 60-day evaluation, 16% of respondents who took at least one dose of antimicrobial prophylaxis (n=842) reported seeking medical care for adverse events caused by prophylaxis at some time during their 60-day course. Nine percent (n=493) reported that their physician or other health-care provider advised them to stop taking antibiotics; 54% of these persons (n=267) reported that the presence of adverse events was the only reason for the recommendation to discontinue. Medical follow-up of persons reporting potentially serious adverse events after 60 days is ongoing.

### Adherence

Fewer than half of respondents (44%, n=2,712) reported taking antimicrobial prophylaxis for at least 60 days. Adherence through 60 days was highest at the Brentwood facility (64%) and lowest at the New York City facility (21%) (Figure). Of persons who took at least one dose of antimicrobial prophylaxis, 72% (n=3,873) reported taking their medicine daily as prescribed, and 19% (n=1,027) reported taking prophylaxis “almost every day.” Eighty-six percent of all respondents were aware of the IND.

**Figure:**
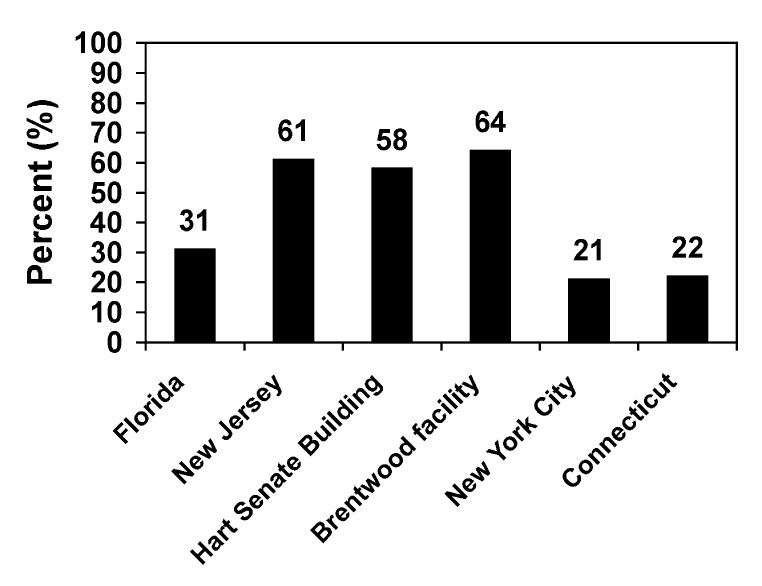
Percentage of persons completing at least 60 days of antimicrobial prophylaxis, by U.S. site, 2001–2002.

Of 2,631 persons taking at least one dose of antimicrobial prophylaxis but stopping before 60 days, 43% stated that adverse events were the most important reason they discontinued prophylaxis, 25% reported perception of a low risk for anthrax, and 7% identified fear of long-term side effects from antimicrobial prophylaxis. Of the 172 who never obtained their prophylaxis, 54% reported perception of a low personal risk for anthrax as the most important reason for not obtaining the recommended antimicrobial agent.

On univariate analysis, in some sites nonadherent respondents were more likely to be African-American, and in other sites they were more likely to be Hispanic. In New York City, nonadherent persons were more likely to have sought medical care and were more likely to have been advised by a health-care provider to stop taking their antimicrobial prophylaxis. In all sites, respondents who enrolled in the IND were more likely to have been adherent. These associations were statistically significant in all but one site (Connecticut) (Table 5).

**Table 5:** Univariate analysis of factors associated with nonadherence, post 60-day program evaluation data, 2001–2002^a^

Variable	Reports of nonadherence^b^ among persons who took at least one dose of postexposure prophylaxis											
	Florida (n=744)		New Jersey (n=1,028)		Hart Senate Building, Washington, D.C. (n = 472)		Brentwood facility, Washington, D.C. (n=1,619)		New York City (n=882)		Connecticut (n=598)	
	(%)	p value	(%)	p value	(%)	p value	(%)	p value	(%)	p value	(%)	p value
**Gender**												
Male	67	0.57	32	<0.01	39	0.71	32	0.27	62	<0.01	68	0.68
Female	69	Ref	47	Ref	41	Ref	34	Ref	77	Ref	70	Ref
**Age group**												
17–39 yr	75	Ref	53	Ref	42	Ref	52	Ref	80	Ref	71	Ref
40–64 yr	61	<0.01	32	<0.01	37	0.33	29	<0.01	67	<0.01	68	0.62
>65 yr	64	0.23	26	<0.01	0	0.08	29	<0.01	33	<0.01	31	<0.01
**Race**												
African-American	82	0.10	44	0.02	53	0.02	33	0.83	72	0.15	72	0.37
All others	67	Ref	35	Ref	38	Ref	32	Ref	67	Ref	68	Ref
**Ethnicity**												
Hispanic	65	0.52	60	<0.01	60	0.19	29	0.67	76	0.03	75	0.30
Non-Hispanic	68	Ref	37	Ref	40	Ref	33	Ref	67	Ref	67	Ref
**Adverse events**												
Yes	75	<0.01	38	0.40	39	0.72	32	0.49	75	<0.01	72	0.13
No	59	Ref	36	Ref	41	Ref	34	Ref	63	Ref	66	Ref
**Severity of adverse events**												
None	64	Ref	37	Ref	45	Ref	33	Ref	63	Ref	65	Ref
Mild	66	0.58	29	0.07	34	0.07	27	0.06	65	0.58	63	0.73
Moderate/ severe	72	0.05	44	0.09	43	0.66	36	0.38	79	<0.01	76	0.01
**IND enrollment**												
Yes	46	<0.01	19	<0.01	7	<0.01	16	<0.01	49	<0.01	60	0.08
No	70	Ref	46	Ref	46	Ref	43	Ref	72	Ref	70	Ref
**Missed ≥1 day of work**												
Yes	72	0.47	40	0.28	33	0.39	32	0.44	77	0.03	74	0.23
No	68	Ref	36	Ref	41	Ref	34	Ref	67	Ref	67	Ref
**Sought medical attention**												
Yes	72	0.36	40	0.42	34	0.18	32	0.58	86	<0.01	65	0.62
No	67	Ref	37	Ref	42	Ref	33	Ref	67	Ref	69	Ref

^a^ IND, investigational new drug. ^b^1–59 days of postexposure prophylaxis.

Six site-specific logistic regression models showed an adverse event to be associated with <60 days’ adherence in two sites only (Florida and New York City). Respondents who enrolled in the IND were more likely to have been adherent in all sites except Connecticut. Hispanic persons were more likely to be nonadherent in New Jersey and New York City; African-American persons were more likely to be nonadherent in New Jersey and the Hart Senate Building site. Persons in the 40- to 64-year age group were less likely to be nonadherent in the Florida, New Jersey, Brentwood, and New York City sites (Table 6).

**Table 6:** Factors associated with nonadherence, multivariable analysis, post 60-day data, 2001–2002^a^

Variable	Participants reporting non-adherence (1–59 days of antimicrobial prophylaxis) among persons taking at least one dose					
	Florida (n=744) OR (95% CI)	New Jersey (n=1,028) OR (95% CI)	Hart Senate Building (n=472) OR (95% CI)	Brentwood facility (n=1,619) OR (95% CI)	New York City (n=882) OR (95% CI)	Connecticut (n=598) OR (95% CI)
**Gender**						
Male	1.00 (0.71, 1.41)	0.56 (0.42, 0.76)	0.92 (0.61, 1.38)	0.90 (0.71, 1.13)	0.57 (0.41, 0.79)	0.99 (0.67, 1.46)
Female	Referent	Referent	Referent	Referent	Referent	Referent
**Age**						
17–39 yr	Referent	Referent	Referent	Referent	Referent	Referent
40–64 yr	0.51 (0.36, 0.72)	0.54 (0.40, 0.73)	0.82 (0.53, 1.28)	0.42 (0.32, 0.56)	0.61 (0.41, 0.90)	0.96 (0.64, 1.44)
>65 yr	0.73 (0.28, 1.89)	0.51 (0.23, 1.12)	n/a	0.49 (0.24, 1.02)	0.17 (0.08, 0.39)	0.21 (0.07, 0.65)
**Race**						
African-American	1.80 (0.66, 4.92)	1.47 (1.06, 2.05)	1.87 (1.08, 3.25)	0.90 (0.63, 1.28)	1.08 (0.76, 1.52)	1.39 (0.86, 2.24)
All others	Referent	Referent	Referent	Referent	Referent	Referent
**Ethnicity**						
Hispanic	0.78 (0.47, 1.29)	2.42 (1.21, 4.86)	1.58 (0.42, 6.01)	0.62 (0.23, 1.69)	1.57 (1.03, 2.39)	1.42 (0.68, 2.93)
Non-Hispanic	Referent	Referent	Referent	Referent	Referent	Referent
**Adverse events**						
Yes	2.23 (1.61, 3.09)	1.08 (0.82, 1.44)	1.07 (0.71, 1.62)	0.98 (0.77, 1.24)	1.58 (1.16, 2.16)	1.32 (0.92, 1.90)
No	Referent	Referent	Referent	Referent	Referent	Referent
**IND enrollment**						
Yes	0.36 (0.21, 0.64)	0.27 (0.19, 0.38)	0.09 (0.04, 0.24)	0.28 (0.22, 0.36)	0.33 (0.21, 0.52)	0.64 (0.39, 1.05)
No	Referent	Referent	Referent	Referent	Referent	Referent

^a^OR, odds ratio; CI, confidence interval; IND, investigational new drug.

## Discussion

 The anthrax outbreak of 2001 represents the first bioterrorist attack in the United States using *B. anthracis* and the first recorded mass postexposure antimicrobial prophylaxis campaign to prevent inhalational anthrax. Monitoring for adverse events and adherence during this campaign offers a unique opportunity to evaluate associated adverse events and adherence to antimicrobial agents in a mass prophylaxis campaign. Our data show that the rate of serious adverse events was low, and adverse event monitoring to date has shown no deaths due to antimicrobial prophylaxis. Mild adverse events or adverse events that did not fulfill criteria as serious were common, and adherence to recommendations for at least 60 days of antimicrobial prophylaxis was poor.

 The overall rate of reported adverse events during this campaign was higher than the rate (16.5%) listed on the usage information provided with ciprofloxacin (13). (The information provided for doxycycline does not include a rate for adverse events, so a similar comparison cannot be made with this agent.) However, comparison of these rates with adverse event rates associated with antimicrobial prophylaxis must be made with caution. Adverse events reported in the ciprofloxacin literature are categorized by their likelihood to be drug related, while this relationship was assessed only for the small proportion of potentially serious adverse events resulting from antimicrobial prophylaxis. Adverse event rates are ideally derived from data collected under controlled circumstances, including the presence of a control group, while these data were collected as part of a response to a public health emergency. Published adverse event rates among patients taking ciprofloxacin or doxycycline in clinical settings where a similar definition of adverse event is used provide a closer comparison of adverse event rates to antimicrobial prophylaxis. A recent published review of adverse events among patients taking long-term (>30 days) ciprofloxacin in clinical trials found an overall rate of 32% and a rate of gastrointestinal adverse events of 22% (14). In several small studies, the rate of adverse events among patients on doxycycline has been shown to be >30% and as high as 50%, with rates of nausea and vomiting of 31%, depending on the reporting method used (15–20).

 Adverse events to antimicrobial prophylaxis in this event may be attributed to the known pharmacology of the drugs taken. However, some portion of the adverse events may also be ascribed to above-average symptom awareness related to fear of contracting anthrax. Data from focus groups of exposed workers support this hypothesis and suggest that self-reports of stress were frequent (21). Anxiety may have led to symptoms or physiologic changes that cannot be explained on the basis of the known pharmacology of the antimicrobial agents given but are temporally related to antimicrobial prophylaxis (22). Regardless of their relation to antimicrobial prophylaxis or fulfillment of criteria for serious adverse events, high rates of reported adverse events during this event suggest the need for a management strategy in addition to monitoring efforts for future antimicrobial prophylaxis campaigns.

While overall adverse events rates were high, differences in rates of adverse events associated with ciprofloxacin compared with those associated with doxycycline were not substantial. Many exposed persons were encouraged to change from ciprofloxacin to doxycycline midway through their course for reasons not related to adverse events (23). Because more than half of persons switched from ciprofloxacin to doxycycline or vice versa, attribution of adverse events to a specific antimicrobial agent is possible only with data collected at the first and second refill visits; adverse event data collected at the program evaluation after 60 days reflect overall adverse events to antimicrobial prophylaxis. Nonetheless, available agent-specific adverse event data do not show differences between ciprofloxacin and doxycycline of the magnitude to warrant preference for one agent over the other in a future antimicrobial prophylaxis campaign.

Overall adherence to recommendations to take at least 60 days of antimicrobial prophylaxis was poor. While adherence to any medical treatment or prophylaxis regimen is essential for treatment to be successful, adherence to antimicrobial prophylaxis is thought to be particularly important because of the risk among persons exposed to *B. anthracis* aerosols for developing anthrax while spores are slowly cleared from lung and thoracic lymphatic systems (4). For this analysis we chose premature discontinuation of antimicrobial prophylaxis as a surrogate for nonadherence, although errors in amount, timing, or frequency can also constitute nonadherence to a medication regimen (24). We found the factor most consistently associated with adherence to be IND participation, which we interpret as a surrogate for perception of individual risk. Because exposed persons were asked to consider their risk for anthrax and the guidance of their health-care provider when making their decision, IND participation is a marker for an person’s perception of risk for inhalational anthrax. Some of the respondents who perceived their risk for anthrax to be high may have been reluctant to enroll in the IND at the end of the initial 60-day regimen because of adverse events in response to antimicrobial prophylaxis, but our univariate analysis did not demonstrate that persons with adverse events were less likely to enroll in the IND protocol. The strong association between risk perception and adherence to antimicrobial prophylaxis is consistent with previous studies of a variety of health conditions, which have demonstrated that effective risk communication based on a close patient-provider relationship is a crucial determinant of adherence (24–26).

 The presence of adverse events was not consistently associated with nonadherence on univariate and multivariable analysis. When asked directly, a higher proportion of nonadherent respondents indicated that the most important reason for their premature discontinuation was adverse events, rather than a low personal risk for anthrax (43% vs. 25%, p<0.01). Of the 1,120 respondents who reported discontinuing antimicrobial prophylaxis because of adverse events, at another point in the interview 16% said that they did not have any adverse events. Despite the fact that many persons recall discontinuing antimicrobial prophylaxis because of adverse events, our analysis showed that risk perception is a stronger and more consistent predictor of adherence across the six exposed cohorts.

Data on adverse events and adherence must be interpreted in light of the unusual circumstances of the bioterrorist attacks of 2001. The difference in clinical and demographic variables between the six sites prevented us from identifying factors related to nonadherence or adverse events for the exposed cohort as a whole. In any future bioterrorist-related *B. anthracis* exposure, site-specific circumstances of the attack and the nature of the exposed population must be taken into account during antimicrobial prophylaxis campaigns. Future adherence promotion activities should consider existing theoretical models developed to predict health behaviors, which often stress the importance of understanding persons’ interest and concern about their health, their perception of the level of risk to their health, and education regarding the consequences of the health problem (27). Adverse event management efforts should help exposed persons manage adverse events regardless of whether they are serious or related to antimicrobial prophylaxis or the terrorism itself. The threat of bioterrorism remains, and we must incorporate lessons learned from the bioterrorist attacks of 2001 to prepare for any future attacks. The data presented here offer public health decision-makers reassurance regarding the low proportion of serious adverse events to antimicrobial prophylaxis and guidance regarding the expected level of adherence during prophylaxis campaigns. Adherence promotion and adverse events management will be essential components to providing this potentially life-saving intervention.
